# Crystal structure of 2-[2-phenyl-1-(phenyl­sulfon­yl)eth­yl]-1-phenyl­sulfonyl-1*H*-indole

**DOI:** 10.1107/S2056989015019428

**Published:** 2015-11-04

**Authors:** M. Umadevi, Potharaju Raju, R. Yamuna, Arasambattu K. Mohanakrishnan, G. Chakkaravarthi

**Affiliations:** a Research and Development Centre, Bharathiyar University, Coimbatore 641 046, India; b Department of Chemistry, Pallavan College of Engineering, Kanchipuram 631 502, India; c Department of Organic Chemistry, University of Madras, Guindy Campus, Chennai 600 025, India; d Department of Sciences, Chemistry and Materials Research Lab, Amrita Vishwa Vidyapeetham University, Ettimadai, Coimbatore 641 112, India; e Department of Physics, CPCL Polytechnic College, Chennai 600 068, India

**Keywords:** crystal structure, indole, hydrogen bonding, C—H⋯π inter­actions

## Abstract

In the title compound, C_28_H_23_NO_4_S_2_, the indole ring system (r.m.s. deviation = 0.007 Å) subtends dihedral angles of 78.69 (13) and 38.97 (13)° with the planes of the N- and C-bonded sulfonyl­benzene rings, respectively, and these two benzene rings are inclined to each other at an angle of 65.45 (16)°. The methyl­ene-linked phenyl ring is twisted at an angle of 81.80 (13)° from the indole ring. The mol­ecular structure features two short intra­molecular C—H⋯O contacts, which both generate *S*(6) rings. In the crystal, mol­ecules are linked by C—H⋯O hydrogen bonds and C—H⋯π inter­actions, generating a three-dimensional network.

## Related literature   

For the biological activity of indole derivatives, see: Chen *et al.* (2015 ▸); Ferro *et al.* (2015 ▸); Parrino *et al.* (2015 ▸); Ma *et al.* (2015 ▸). For a related structure, see: Umadevi *et al.* (2015 ▸).
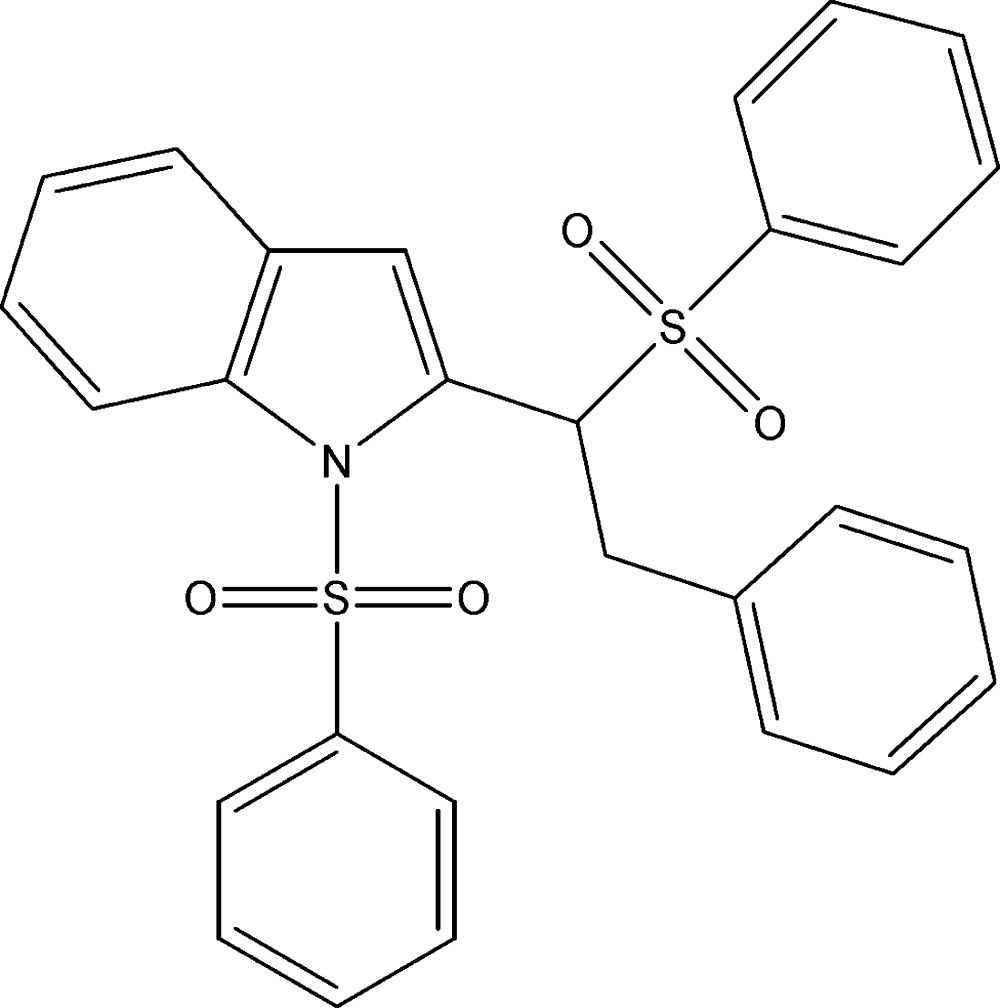


## Experimental   

### Crystal data  

C_28_H_23_NO_4_S_2_
*M* *_r_* = 501.59
Triclinic, 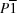

*a* = 9.7485 (6) Å
*b* = 10.4930 (6) Å
*c* = 12.2879 (6) Å
α = 94.479 (3)°
β = 96.926 (3)°
γ = 102.253 (3)°
*V* = 1212.36 (12) Å^3^
*Z* = 2
Mo *K*α radiation
μ = 0.26 mm^−1^
*T* = 295 K
0.28 × 0.24 × 0.22 mm

### Data collection  

Bruker Kappa APEXII CCD diffractometer
Absorption correction: multi-scan (*SADABS*; Sheldrick, 1996 ▸) *T*_min_ = 0.932, *T*_max_ = 0.946
28630 measured reflections
5104 independent reflections
3744 reflections with *I* > 2σ(*I*)
*R*_int_ = 0.030

### Refinement  

*R*[*F* ^2^ > 2σ(*F* ^2^)] = 0.049
*wR*(*F* ^2^) = 0.109
*S* = 1.07
5104 reflections
316 parameters
H-atom parameters constrained
Δρ_max_ = 0.34 e Å^−3^
Δρ_min_ = −0.58 e Å^−3^

### 

Data collection: *APEX2* (Bruker, 2004 ▸); cell refinement: *SAINT* (Bruker, 2004 ▸); data reduction: *SAINT*; program(s) used to solve structure: *SHELXS97* (Sheldrick, 2008 ▸); program(s) used to refine structure: *SHELXL97* (Sheldrick, 2008 ▸); molecular graphics: *PLATON* (Spek, 2009 ▸); software used to prepare material for publication: *SHELXL97* (Sheldrick, 2008 ▸) and *PLATON*.

## Supplementary Material

Crystal structure: contains datablock(s) global, I. DOI: 10.1107/S2056989015019428/hb7517sup1.cif

Structure factors: contains datablock(s) I. DOI: 10.1107/S2056989015019428/hb7517Isup2.hkl

Supporting information file. DOI: 10.1107/S2056989015019428/hb7517Isup3.cml

**Fig. 1.**
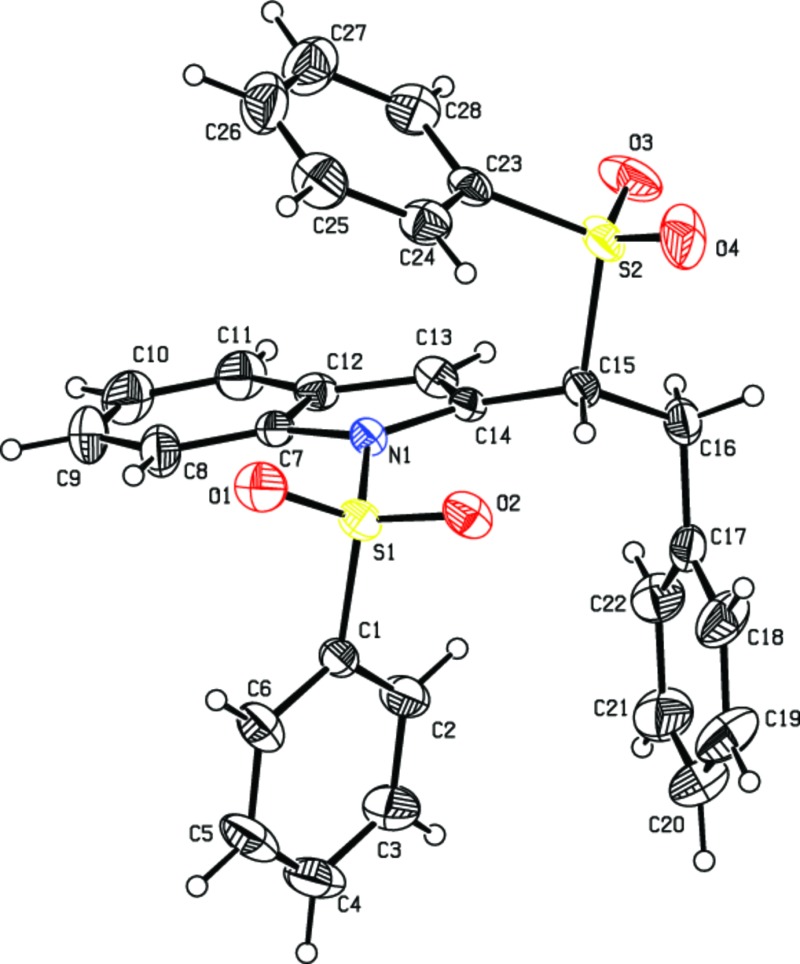
The molecular structure of the title compound, with displacement ellipsoids drawn at the 30% probability level.

**Fig. 2.**
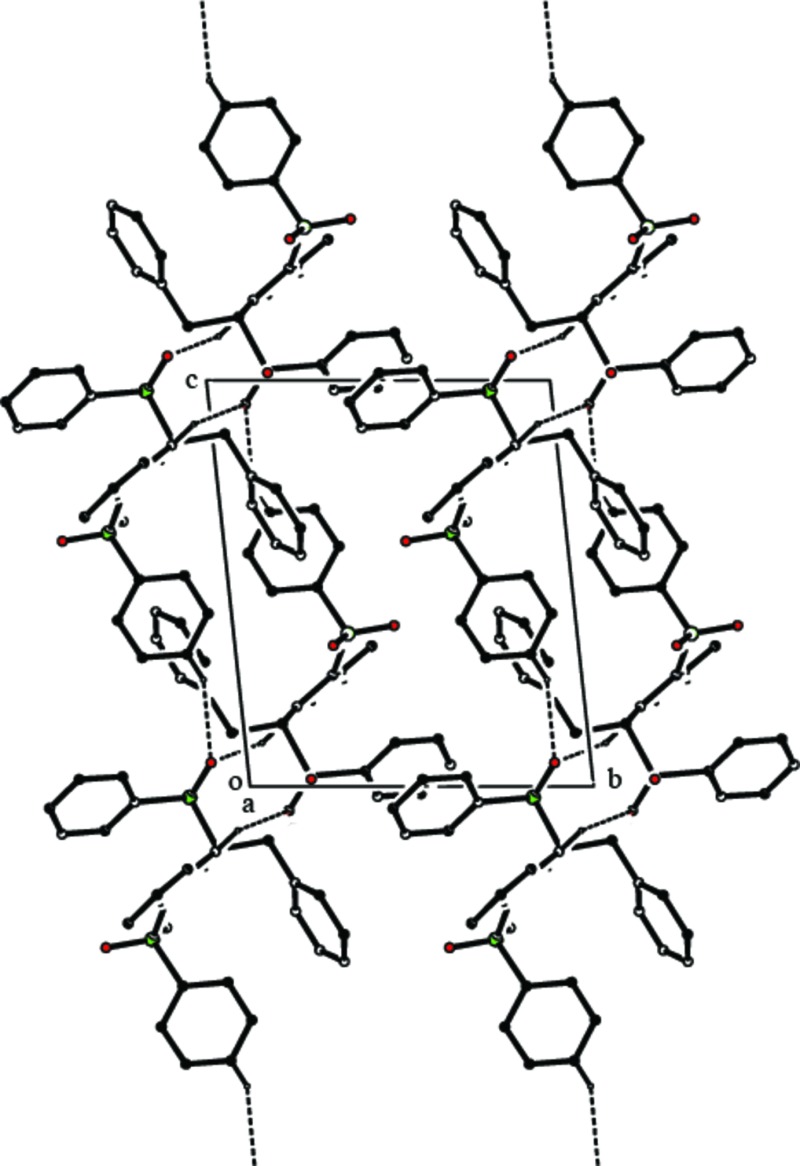
The crystal packing of the title compound, viewed along the a axis. The C—H···O hydrogen bonds are shown as dashed lines (see Table 1). H atoms not involved in these interactions have been omitted for clarity.

. DOI: 10.1107/S2056989015019428/hb7517fig1.tif
The mol­ecular structure of the title compound, with displacement ellipsoids drawn at the 30% probability level.

a . DOI: 10.1107/S2056989015019428/hb7517fig2.tif
The crystal packing of the title compound, viewed along the *a* axis. The C—H⋯O hydrogen bonds are shown as dashed lines (see Table 1). H atoms not involved in these inter­actions have been omitted for clarity.

CCDC reference: 1431246

Additional supporting information:  crystallographic information; 3D view; checkCIF report

## Figures and Tables

**Table 1:** Hydrogen-bond geometry (Å, °) *Cg*2 and *Cg*4 are the centroids of the C1–C6 and C17–C22 rings, respectively.

*D*—H⋯*A*	*D*—H	H⋯*A*	*D*⋯*A*	*D*—H⋯*A*
C8—H8⋯O1	0.93	2.43	3.009 (4)	121
C15—H15⋯O2	0.98	2.11	2.907 (3)	137
C4—H4⋯O3^i^	0.93	2.49	3.314 (3)	148
C13—H13⋯O3^ii^	0.93	2.50	3.240 (3)	137
C2—H2⋯*Cg*4	0.93	2.83	3.544 (3)	134
C19—H19⋯*Cg*2^iii^	0.93	2.91	3.620 (4)	134

Symmetry codes: (i) 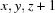; (ii) 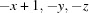; (iii) 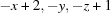.

## supplementary crystallographic information

### S1. Structural commentary

Indole derivatives are known to exhibit biological activities such as anti-proliferative (Parrino *et al.*, 2015), potential mushroom tyrosinase inhibitors (Ferro *et al.*, 2015), anti-inflammatory (Chen *et al.*, 2015) and anti-tumor (Ma *et al.*, 2015). We herein report the crystal structure of (I) (Fig. 1). The geometric parameters of the title compound agree well with a similar structure [Umadevi *et al.* 2015].

The indole moiety is almost planar (r.m.s deviation = 0.007 Å] and makes the dihedral angles of 78.69 (13)° with N-bound phenyl­sulfonyl ring (C1—C6), 38.97 (13)° with C-bound phenyl­sulfonyl ring (C23—C28) and 81.80 (13)° with phenyl ring (C17—C22). The N-bound and C-bound phenyl­sulfonyl rings are inclined at an angle of 65.45 (16)°. The molecular structure is stabilized by weak intra­molecular C—H···O hydrogen bond and C—H···π (Table 1) inter­action. The crystal structure is formed by weak inter­molecular C—H···O hydrogen bonds (Fig. 2 & Table 1) and C—H···π (Table 1) inter­actions.

### S2. Synthesis and crystallization

To a solution of 1-(phenyl­sulfonyl)-2-(phenyl­sulfonyl­methyl)-1H-indole (0.5 g, 1.21 mmol) in dry DMF (10 ml) K_2_CO_3_ (0.33 g, 2.43 mmol) and benzyl chloride (0.20 g, 1.58 mmol) were added and the reaction mixture was stirred at room temperature for 12 h. after completion of starting material (monitored by TLC), the reaction mass was poured over crushed ice containing Conc. HCl (3 ml) and extracted with ethyl acetate (20 ml). The combined organic extracts were washed with water (3 ml), brine solution (3 ml) and dried (Na_2_SO_4_). Removal of solvent followed by recrystallization of the crude product from methanol (5 ml) afforded the title compound as colourless blocks.

### S3. Refinement

H atoms were positioned geometrically and refined using riding model, with C—H = 0.93 Å and *U*_iso_(H) = 1.2Ueq(C) for aromatic CH, C—H = 0.98 Å and *U*_iso_(H) = 1.2Ueq(C) for CH and C—H = 0.97 Å and *U*_iso_(H) = 1.2Ueq(C) for CH_2_.

### Crystal data

**Table:**

C_28_H_23_NO_4_S_2_	*Z* = 2
*M**_r_* = 501.59	*F*(000) = 524
Triclinic, *P*1	*D*_x_ = 1.374 Mg m^−^^3^
Hall symbol: -P 1	Mo *K*α radiation, λ = 0.71073 Å
*a* = 9.7485 (6) Å	Cell parameters from 9872 reflections
*b* = 10.4930 (6) Å	θ = 2.2–26.6°
*c* = 12.2879 (6) Å	µ = 0.26 mm^−^^1^
α = 94.479 (3)°	*T* = 295 K
β = 96.926 (3)°	Block, colourless
γ = 102.253 (3)°	0.28 × 0.24 × 0.22 mm
*V* = 1212.36 (12) Å^3^	

### Data collection

**Table:**

Bruker Kappa APEXII CCD diffractometer	5104 independent reflections
Radiation source: fine-focus sealed tube	3744 reflections with *I* > 2σ(*I*)
Graphite monochromator	*R*_int_ = 0.030
ω and φ scan	θ_max_ = 26.8°, θ_min_ = 2.0°
Absorption correction: multi-scan (*SADABS*; Sheldrick, 1996)	*h* = −12→12
*T*_min_ = 0.932, *T*_max_ = 0.946	*k* = −13→13
28630 measured reflections	*l* = −15→15

### Refinement

**Table:**

Refinement on *F*^2^	Primary atom site location: structure-invariant direct methods
Least-squares matrix: full	Secondary atom site location: difference Fourier map
*R*[*F*^2^ > 2σ(*F*^2^)] = 0.049	Hydrogen site location: inferred from neighbouring sites
*wR*(*F*^2^) = 0.109	H-atom parameters constrained
*S* = 1.07	*w* = 1/[σ^2^(*F*_o_^2^) + (0.0176*P*)^2^ + 1.3407*P*] where *P* = (*F*_o_^2^ + 2*F*_c_^2^)/3
5104 reflections	(Δ/σ)_max_ < 0.001
316 parameters	Δρ_max_ = 0.34 e Å^−^^3^
0 restraints	Δρ_min_ = −0.58 e Å^−^^3^

### Special details

**Table:**

Geometry. All esds (except the esd in the dihedral angle between two l.s. planes) are estimated using the full covariance matrix. The cell esds are taken into account individually in the estimation of esds in distances, angles and torsion angles; correlations between esds in cell parameters are only used when they are defined by crystal symmetry. An approximate (isotropic) treatment of cell esds is used for estimating esds involving l.s. planes.
Refinement. Refinement of F^2^ against ALL reflections. The weighted R-factor wR and goodness of fit S are based on F^2^, conventional R-factors R are based on F, with F set to zero for negative F^2^. The threshold expression of F^2^ > 2sigma(F^2^) is used only for calculating R-factors(gt) etc. and is not relevant to the choice of reflections for refinement. R-factors based on F^2^ are statistically about twice as large as those based on F, and R- factors based on ALL data will be even larger.

### Fractional atomic coordinates and isotropic or equivalent isotropic displacement parameters (Å^2^)

**Table:**

	*x*	*y*	*z*	*U*_iso_*/*U*_eq_	
C1	0.7800 (2)	0.2579 (2)	0.49099 (18)	0.0381 (5)	
C2	0.7195 (3)	0.1253 (3)	0.4812 (2)	0.0536 (7)	
H2	0.7028	0.0754	0.4130	0.064*	
C3	0.6844 (4)	0.0681 (3)	0.5743 (3)	0.0672 (8)	
H3	0.6431	−0.0210	0.5690	0.081*	
C4	0.7100 (4)	0.1417 (4)	0.6744 (2)	0.0721 (10)	
H4	0.6871	0.1024	0.7370	0.087*	
C5	0.7693 (4)	0.2735 (4)	0.6827 (2)	0.0719 (10)	
H5	0.7856	0.3229	0.7511	0.086*	
C6	0.8052 (3)	0.3336 (3)	0.5911 (2)	0.0551 (7)	
H6	0.8454	0.4230	0.5967	0.066*	
C7	0.5685 (2)	0.2997 (2)	0.28275 (18)	0.0364 (5)	
C8	0.5302 (3)	0.3979 (3)	0.3462 (2)	0.0565 (7)	
H8	0.5973	0.4611	0.3931	0.068*	
C9	0.3882 (4)	0.3974 (3)	0.3363 (3)	0.0703 (9)	
H9	0.3585	0.4615	0.3782	0.084*	
C10	0.2879 (3)	0.3042 (4)	0.2659 (3)	0.0677 (9)	
H10	0.1928	0.3073	0.2613	0.081*	
C11	0.3263 (3)	0.2080 (3)	0.2032 (2)	0.0553 (7)	
H11	0.2586	0.1460	0.1558	0.066*	
C12	0.4692 (2)	0.2047 (2)	0.21191 (19)	0.0391 (5)	
C13	0.5447 (2)	0.1211 (2)	0.15991 (19)	0.0395 (5)	
H13	0.5040	0.0496	0.1080	0.047*	
C14	0.6838 (2)	0.1612 (2)	0.19720 (17)	0.0321 (5)	
C15	0.8030 (3)	0.1136 (2)	0.15503 (18)	0.0372 (5)	
H15	0.8880	0.1440	0.2097	0.045*	
C16	0.7758 (3)	−0.0364 (2)	0.1323 (2)	0.0487 (6)	
H16A	0.6854	−0.0690	0.0857	0.058*	
H16B	0.8492	−0.0592	0.0930	0.058*	
C17	0.7744 (3)	−0.1007 (2)	0.2368 (2)	0.0464 (6)	
C18	0.8982 (3)	−0.0935 (3)	0.3066 (3)	0.0713 (9)	
H18	0.9838	−0.0485	0.2883	0.086*	
C19	0.8969 (4)	−0.1517 (4)	0.4026 (3)	0.0884 (12)	
H19	0.9814	−0.1445	0.4492	0.106*	
C20	0.7738 (5)	−0.2196 (4)	0.4301 (3)	0.0833 (11)	
H20	0.7741	−0.2610	0.4943	0.100*	
C21	0.6501 (4)	−0.2271 (3)	0.3636 (3)	0.0746 (10)	
H21	0.5652	−0.2719	0.3832	0.089*	
C22	0.6499 (3)	−0.1682 (3)	0.2671 (2)	0.0588 (7)	
H22	0.5646	−0.1742	0.2218	0.071*	
C23	0.8138 (3)	0.3404 (2)	0.03825 (19)	0.0419 (6)	
C24	0.9082 (3)	0.4365 (3)	0.1084 (2)	0.0544 (7)	
H24	0.9866	0.4176	0.1494	0.065*	
C25	0.8843 (4)	0.5624 (3)	0.1169 (3)	0.0716 (9)	
H25	0.9450	0.6280	0.1661	0.086*	
C26	0.7725 (4)	0.5902 (3)	0.0535 (3)	0.0803 (11)	
H26	0.7580	0.6751	0.0589	0.096*	
C27	0.6815 (4)	0.4954 (4)	−0.0176 (3)	0.0806 (10)	
H27	0.6067	0.5161	−0.0619	0.097*	
C28	0.7000 (3)	0.3684 (3)	−0.0244 (3)	0.0619 (8)	
H28	0.6359	0.3025	−0.0710	0.074*	
N1	0.70401 (19)	0.27497 (18)	0.27447 (14)	0.0333 (4)	
O1	0.8486 (2)	0.46961 (17)	0.39621 (15)	0.0570 (5)	
O2	0.95665 (17)	0.28679 (19)	0.34733 (14)	0.0507 (5)	
O3	0.7321 (3)	0.10587 (19)	−0.05895 (15)	0.0762 (7)	
O4	0.9842 (3)	0.1806 (2)	0.0183 (2)	0.0821 (8)	
S1	0.83746 (6)	0.33264 (6)	0.37640 (5)	0.03940 (15)	
S2	0.83884 (8)	0.17893 (6)	0.02663 (5)	0.04973 (19)	

### Atomic displacement parameters (Å^2^)

**Table:**

	*U*^11^	*U*^22^	*U*^33^	*U*^12^	*U*^13^	*U*^23^
C1	0.0340 (12)	0.0513 (14)	0.0312 (11)	0.0156 (11)	0.0035 (9)	0.0023 (10)
C2	0.0675 (19)	0.0548 (16)	0.0382 (14)	0.0107 (14)	0.0107 (13)	0.0056 (12)
C3	0.077 (2)	0.072 (2)	0.0570 (18)	0.0150 (17)	0.0192 (16)	0.0214 (16)
C4	0.066 (2)	0.117 (3)	0.0420 (16)	0.029 (2)	0.0174 (15)	0.0277 (18)
C5	0.068 (2)	0.115 (3)	0.0307 (14)	0.021 (2)	0.0085 (13)	−0.0044 (16)
C6	0.0494 (16)	0.0736 (19)	0.0405 (14)	0.0146 (14)	0.0069 (12)	−0.0086 (13)
C7	0.0376 (13)	0.0438 (13)	0.0325 (11)	0.0155 (10)	0.0081 (9)	0.0096 (10)
C8	0.0579 (18)	0.0594 (17)	0.0569 (17)	0.0269 (14)	0.0088 (14)	−0.0034 (13)
C9	0.072 (2)	0.080 (2)	0.076 (2)	0.0471 (19)	0.0254 (18)	0.0069 (18)
C10	0.0442 (17)	0.093 (2)	0.079 (2)	0.0337 (17)	0.0154 (16)	0.0245 (19)
C11	0.0361 (14)	0.0706 (19)	0.0612 (17)	0.0141 (13)	0.0068 (12)	0.0138 (15)
C12	0.0326 (12)	0.0494 (14)	0.0365 (12)	0.0084 (10)	0.0072 (10)	0.0110 (10)
C13	0.0374 (13)	0.0417 (13)	0.0357 (12)	0.0016 (10)	0.0060 (10)	0.0005 (10)
C14	0.0382 (12)	0.0329 (11)	0.0269 (10)	0.0084 (9)	0.0090 (9)	0.0053 (9)
C15	0.0406 (13)	0.0378 (12)	0.0351 (12)	0.0103 (10)	0.0118 (10)	0.0016 (10)
C16	0.0622 (17)	0.0389 (13)	0.0497 (15)	0.0161 (12)	0.0205 (13)	0.0010 (11)
C17	0.0542 (16)	0.0372 (13)	0.0540 (15)	0.0201 (12)	0.0144 (12)	0.0044 (11)
C18	0.0500 (18)	0.074 (2)	0.101 (3)	0.0284 (16)	0.0145 (17)	0.0323 (19)
C19	0.082 (3)	0.091 (3)	0.101 (3)	0.041 (2)	−0.008 (2)	0.037 (2)
C20	0.113 (3)	0.068 (2)	0.081 (2)	0.035 (2)	0.017 (2)	0.0373 (19)
C21	0.083 (2)	0.0584 (19)	0.085 (2)	0.0074 (17)	0.024 (2)	0.0262 (18)
C22	0.0602 (18)	0.0491 (16)	0.0636 (18)	0.0035 (14)	0.0073 (14)	0.0107 (14)
C23	0.0491 (15)	0.0422 (13)	0.0344 (12)	0.0027 (11)	0.0176 (11)	0.0066 (10)
C24	0.0609 (18)	0.0486 (16)	0.0498 (16)	0.0019 (13)	0.0098 (13)	0.0077 (12)
C25	0.095 (3)	0.0418 (16)	0.071 (2)	−0.0038 (17)	0.0230 (19)	0.0002 (15)
C26	0.096 (3)	0.054 (2)	0.103 (3)	0.027 (2)	0.042 (2)	0.017 (2)
C27	0.068 (2)	0.089 (3)	0.099 (3)	0.036 (2)	0.022 (2)	0.031 (2)
C28	0.0578 (18)	0.0658 (19)	0.0601 (18)	0.0080 (15)	0.0097 (15)	0.0074 (15)
N1	0.0338 (10)	0.0360 (10)	0.0296 (9)	0.0078 (8)	0.0050 (8)	0.0000 (8)
O1	0.0690 (13)	0.0390 (10)	0.0527 (11)	−0.0034 (9)	0.0004 (9)	−0.0035 (8)
O2	0.0323 (9)	0.0753 (13)	0.0435 (10)	0.0100 (9)	0.0073 (7)	0.0017 (9)
O3	0.128 (2)	0.0516 (12)	0.0348 (10)	−0.0095 (12)	0.0162 (11)	−0.0058 (9)
O4	0.0838 (16)	0.0688 (14)	0.1168 (19)	0.0287 (12)	0.0735 (15)	0.0273 (13)
S1	0.0369 (3)	0.0433 (3)	0.0340 (3)	0.0025 (3)	0.0040 (2)	−0.0011 (2)
S2	0.0668 (5)	0.0409 (3)	0.0434 (4)	0.0055 (3)	0.0289 (3)	0.0016 (3)

### Geometric parameters (Å, º)

**Table:**

C1—C6	1.376 (3)	C16—C17	1.497 (4)
C1—C2	1.381 (4)	C16—H16A	0.9700
C1—S1	1.757 (2)	C16—H16B	0.9700
C2—C3	1.379 (4)	C17—C18	1.377 (4)
C2—H2	0.9300	C17—C22	1.378 (4)
C3—C4	1.366 (4)	C18—C19	1.370 (5)
C3—H3	0.9300	C18—H18	0.9300
C4—C5	1.371 (5)	C19—C20	1.355 (5)
C4—H4	0.9300	C19—H19	0.9300
C5—C6	1.379 (4)	C20—C21	1.356 (5)
C5—H5	0.9300	C20—H20	0.9300
C6—H6	0.9300	C21—C22	1.380 (4)
C7—C8	1.385 (3)	C21—H21	0.9300
C7—C12	1.392 (3)	C22—H22	0.9300
C7—N1	1.413 (3)	C23—C28	1.370 (4)
C8—C9	1.374 (4)	C23—C24	1.373 (4)
C8—H8	0.9300	C23—S2	1.760 (3)
C9—C10	1.385 (5)	C24—C25	1.387 (4)
C9—H9	0.9300	C24—H24	0.9300
C10—C11	1.365 (4)	C25—C26	1.358 (5)
C10—H10	0.9300	C25—H25	0.9300
C11—C12	1.392 (3)	C26—C27	1.357 (5)
C11—H11	0.9300	C26—H26	0.9300
C12—C13	1.421 (3)	C27—C28	1.379 (5)
C13—C14	1.343 (3)	C27—H27	0.9300
C13—H13	0.9300	C28—H28	0.9300
C14—N1	1.429 (3)	N1—S1	1.6679 (18)
C14—C15	1.490 (3)	O1—S1	1.4176 (18)
C15—C16	1.536 (3)	O2—S1	1.4211 (18)
C15—S2	1.812 (2)	O3—S2	1.431 (2)
C15—H15	0.9800	O4—S2	1.430 (2)
			
C6—C1—C2	121.5 (2)	H16A—C16—H16B	108.0
C6—C1—S1	118.0 (2)	C18—C17—C22	117.7 (3)
C2—C1—S1	120.35 (18)	C18—C17—C16	120.9 (3)
C3—C2—C1	118.9 (3)	C22—C17—C16	121.4 (3)
C3—C2—H2	120.6	C19—C18—C17	120.9 (3)
C1—C2—H2	120.6	C19—C18—H18	119.5
C4—C3—C2	120.3 (3)	C17—C18—H18	119.5
C4—C3—H3	119.9	C20—C19—C18	120.6 (3)
C2—C3—H3	119.9	C20—C19—H19	119.7
C3—C4—C5	120.2 (3)	C18—C19—H19	119.7
C3—C4—H4	119.9	C19—C20—C21	119.8 (3)
C5—C4—H4	119.9	C19—C20—H20	120.1
C4—C5—C6	120.8 (3)	C21—C20—H20	120.1
C4—C5—H5	119.6	C20—C21—C22	120.1 (3)
C6—C5—H5	119.6	C20—C21—H21	119.9
C1—C6—C5	118.3 (3)	C22—C21—H21	119.9
C1—C6—H6	120.9	C17—C22—C21	120.8 (3)
C5—C6—H6	120.9	C17—C22—H22	119.6
C8—C7—C12	122.3 (2)	C21—C22—H22	119.6
C8—C7—N1	129.9 (2)	C28—C23—C24	120.9 (3)
C12—C7—N1	107.88 (19)	C28—C23—S2	119.0 (2)
C9—C8—C7	116.6 (3)	C24—C23—S2	120.1 (2)
C9—C8—H8	121.7	C23—C24—C25	118.7 (3)
C7—C8—H8	121.7	C23—C24—H24	120.7
C8—C9—C10	122.0 (3)	C25—C24—H24	120.7
C8—C9—H9	119.0	C26—C25—C24	120.2 (3)
C10—C9—H9	119.0	C26—C25—H25	119.9
C11—C10—C9	121.1 (3)	C24—C25—H25	119.9
C11—C10—H10	119.4	C27—C26—C25	120.8 (3)
C9—C10—H10	119.4	C27—C26—H26	119.6
C10—C11—C12	118.5 (3)	C25—C26—H26	119.6
C10—C11—H11	120.7	C26—C27—C28	120.1 (3)
C12—C11—H11	120.7	C26—C27—H27	120.0
C11—C12—C7	119.5 (2)	C28—C27—H27	120.0
C11—C12—C13	133.2 (2)	C23—C28—C27	119.3 (3)
C7—C12—C13	107.3 (2)	C23—C28—H28	120.4
C14—C13—C12	109.5 (2)	C27—C28—H28	120.4
C14—C13—H13	125.2	C7—N1—C14	106.97 (18)
C12—C13—H13	125.2	C7—N1—S1	120.12 (15)
C13—C14—N1	108.34 (19)	C14—N1—S1	128.16 (15)
C13—C14—C15	127.7 (2)	O1—S1—O2	118.97 (12)
N1—C14—C15	123.26 (19)	O1—S1—N1	107.18 (11)
C14—C15—C16	114.0 (2)	O2—S1—N1	107.21 (10)
C14—C15—S2	110.79 (16)	O1—S1—C1	108.76 (11)
C16—C15—S2	106.56 (15)	O2—S1—C1	109.44 (11)
C14—C15—H15	108.4	N1—S1—C1	104.28 (10)
C16—C15—H15	108.4	O4—S2—O3	118.72 (15)
S2—C15—H15	108.4	O4—S2—C23	109.84 (12)
C17—C16—C15	111.6 (2)	O3—S2—C23	106.77 (14)
C17—C16—H16A	109.3	O4—S2—C15	106.67 (13)
C15—C16—H16A	109.3	O3—S2—C15	107.65 (11)
C17—C16—H16B	109.3	C23—S2—C15	106.58 (10)
C15—C16—H16B	109.3		
			
C6—C1—C2—C3	−0.2 (4)	S2—C23—C24—C25	−179.1 (2)
S1—C1—C2—C3	175.7 (2)	C23—C24—C25—C26	−2.5 (5)
C1—C2—C3—C4	−0.4 (5)	C24—C25—C26—C27	0.9 (5)
C2—C3—C4—C5	0.8 (5)	C25—C26—C27—C28	1.5 (6)
C3—C4—C5—C6	−0.6 (5)	C24—C23—C28—C27	0.8 (4)
C2—C1—C6—C5	0.4 (4)	S2—C23—C28—C27	−178.5 (2)
S1—C1—C6—C5	−175.5 (2)	C26—C27—C28—C23	−2.4 (5)
C4—C5—C6—C1	0.0 (5)	C8—C7—N1—C14	179.9 (2)
C12—C7—C8—C9	0.2 (4)	C12—C7—N1—C14	−0.6 (2)
N1—C7—C8—C9	179.6 (3)	C8—C7—N1—S1	22.4 (3)
C7—C8—C9—C10	−0.5 (5)	C12—C7—N1—S1	−158.08 (16)
C8—C9—C10—C11	0.2 (5)	C13—C14—N1—C7	0.9 (2)
C9—C10—C11—C12	0.4 (5)	C15—C14—N1—C7	171.90 (19)
C10—C11—C12—C7	−0.7 (4)	C13—C14—N1—S1	156.01 (17)
C10—C11—C12—C13	−179.6 (3)	C15—C14—N1—S1	−33.0 (3)
C8—C7—C12—C11	0.4 (4)	C7—N1—S1—O1	−54.17 (19)
N1—C7—C12—C11	−179.1 (2)	C14—N1—S1—O1	153.59 (18)
C8—C7—C12—C13	179.6 (2)	C7—N1—S1—O2	177.05 (17)
N1—C7—C12—C13	0.1 (2)	C14—N1—S1—O2	24.8 (2)
C11—C12—C13—C14	179.5 (3)	C7—N1—S1—C1	61.05 (19)
C7—C12—C13—C14	0.5 (3)	C14—N1—S1—C1	−91.20 (19)
C12—C13—C14—N1	−0.9 (3)	C6—C1—S1—O1	−23.8 (2)
C12—C13—C14—C15	−171.4 (2)	C2—C1—S1—O1	160.2 (2)
C13—C14—C15—C16	−44.7 (3)	C6—C1—S1—O2	107.6 (2)
N1—C14—C15—C16	146.1 (2)	C2—C1—S1—O2	−68.4 (2)
C13—C14—C15—S2	75.5 (3)	C6—C1—S1—N1	−137.9 (2)
N1—C14—C15—S2	−93.7 (2)	C2—C1—S1—N1	46.1 (2)
C14—C15—C16—C17	−69.2 (3)	C28—C23—S2—O4	134.5 (2)
S2—C15—C16—C17	168.22 (19)	C24—C23—S2—O4	−44.9 (2)
C15—C16—C17—C18	−72.9 (3)	C28—C23—S2—O3	4.5 (2)
C15—C16—C17—C22	106.7 (3)	C24—C23—S2—O3	−174.8 (2)
C22—C17—C18—C19	0.1 (5)	C28—C23—S2—C15	−110.3 (2)
C16—C17—C18—C19	179.6 (3)	C24—C23—S2—C15	70.3 (2)
C17—C18—C19—C20	1.1 (6)	C14—C15—S2—O4	154.36 (17)
C18—C19—C20—C21	−1.9 (6)	C16—C15—S2—O4	−81.1 (2)
C19—C20—C21—C22	1.5 (6)	C14—C15—S2—O3	−77.20 (19)
C18—C17—C22—C21	−0.4 (4)	C16—C15—S2—O3	47.4 (2)
C16—C17—C22—C21	−180.0 (3)	C14—C15—S2—C23	37.04 (19)
C20—C21—C22—C17	−0.3 (5)	C16—C15—S2—C23	161.59 (17)
C28—C23—C24—C25	1.6 (4)		

### Hydrogen-bond geometry (Å, º)

Cg2 and Cg4 are the centroids of the C1–C6 and C17–C22 rings, respectively.

**Table:**

*D*—H···*A*	*D*—H	H···*A*	*D*···*A*	*D*—H···*A*
C8—H8···O1	0.93	2.43	3.009 (4)	121
C15—H15···O2	0.98	2.11	2.907 (3)	137
C4—H4···O3^i^	0.93	2.49	3.314 (3)	148
C13—H13···O3^ii^	0.93	2.50	3.240 (3)	137
C2—H2···*Cg*4	0.93	2.83	3.544 (3)	134
C19—H19···*Cg*2^iii^	0.93	2.91	3.620 (4)	134

Symmetry codes: (i) *x*, *y*, *z*+1; (ii) −*x*+1, −*y*, −*z*; (iii) −*x*+2, −*y*, −*z*+1.
