# Supplementary figures and images for: Crystal structure of 2-[2-phenyl-1-(phenyl­sulfon­yl)eth­yl]-1-phenyl­sulfonyl-1H-indole
Source: Acta Crystallogr E Crystallogr Commun. 2015 Nov 4;71(Pt 12):o910–1. doi: 10.1107/S2056989015019428 (PMC4719875; doi:10.1107/S2056989015019428)

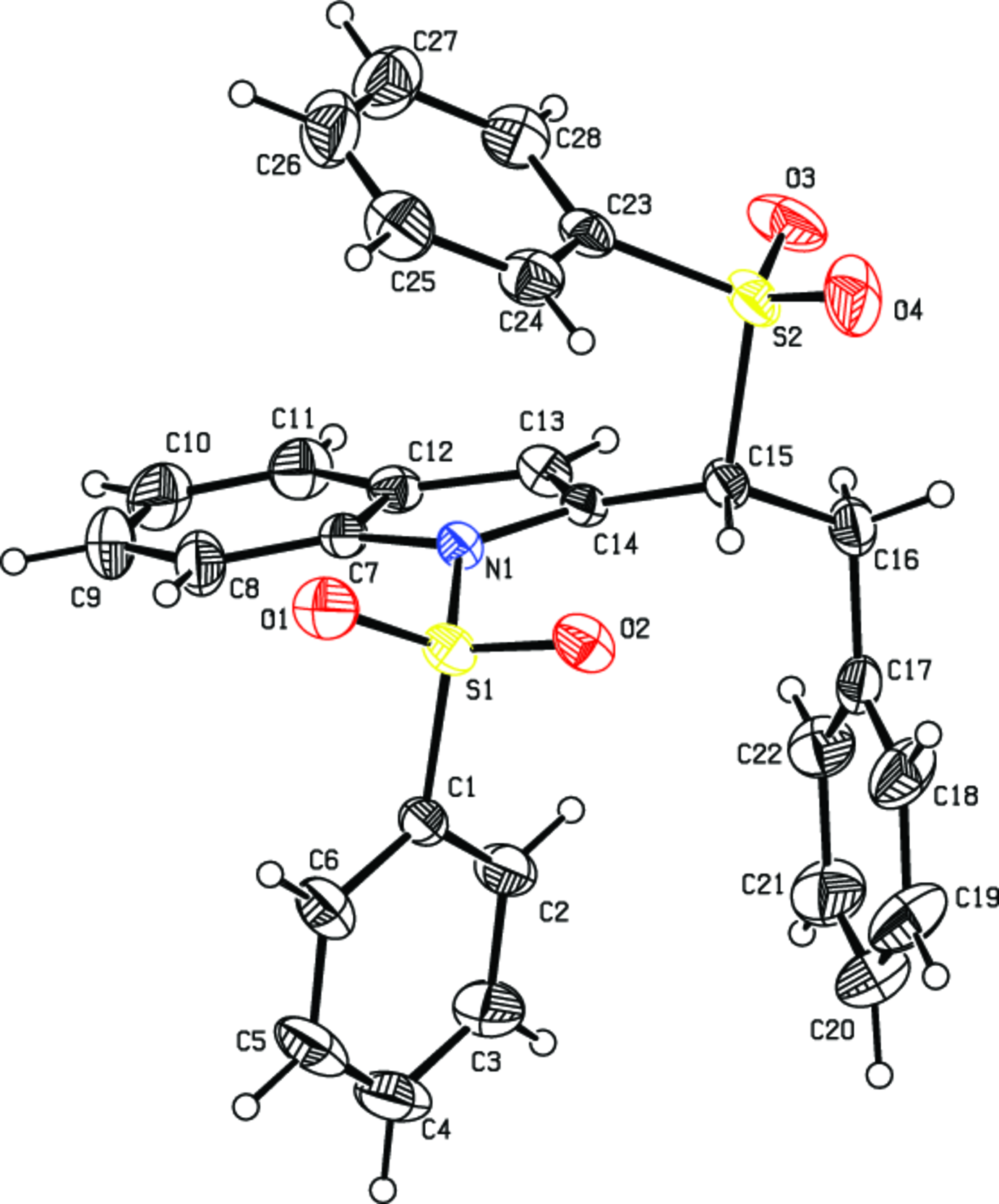

Supplement: Supplementary file 4 [file e-71-0o910-fig1.tif]

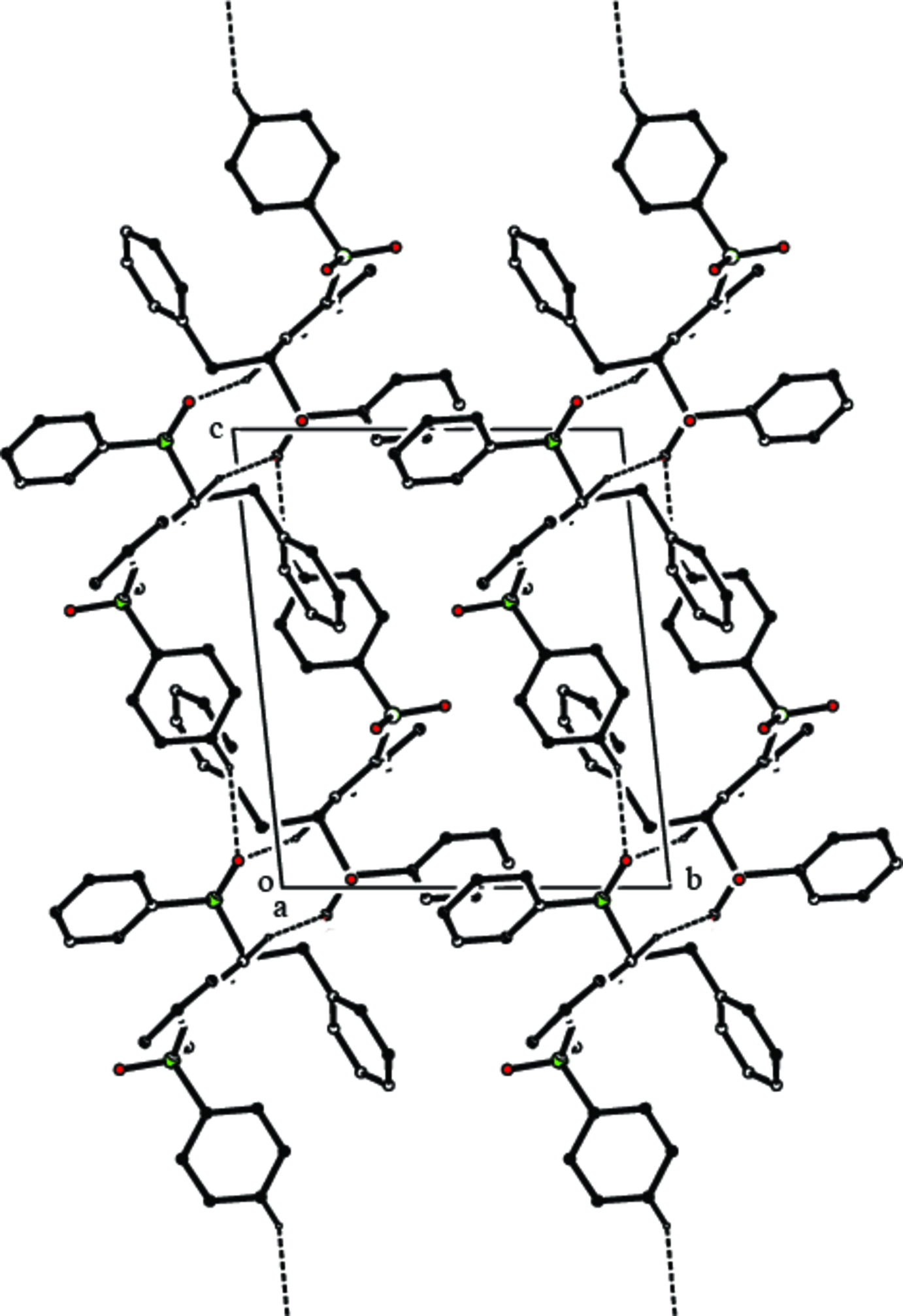

Supplement: Supplementary file 5 [file e-71-0o910-fig2.tif]
